# Citrate-based dietary alkali supplements available in Germany: an overview

**DOI:** 10.1186/s40780-024-00342-0

**Published:** 2024-05-10

**Authors:** Maximilian Andreas Storz, Alvaro Luis Ronco

**Affiliations:** 1 Department of Internal Medicine II, Centre for Complementary Medicine, Faculty of Medicine, Medical Center – University of Freiburg, Freiburg, Germany; 2 Unit of Oncology and Radiotherapy, Pereira Rossell Women’s Hospital, Bvard. Artigas 1590, Montevideo, 11600 Uruguay; 3 Biomedical Sciences Center, University of Montevideo, Puntas de Santiago 1604, Montevideo, 11500 Uruguay

**Keywords:** Dietary supplements, Potential renal acid load, Minerals, Vitamins, Citrates, Acid-base equilibrium

## Abstract

**Background:**

Fruits and vegetables are abundant in alkali precursors and effectively reduce the Potential Renal Acid Load (PRAL) from diet. Oral alkali supplements are supposed to exert comparable alkalizing effects on the human body, and have been shown to beneficially affect bone and kidney health. A comparative analysis of the available dietary alkali supplements in Germany was performed, contrasting their potential PRAL-lowering potential.

**Methods:**

We reviewed the currently available dietary citrate-based alkali supplements sold in Germany with a special focus on their mineral content, their PRAL-lowering potential and other characteristics inherent to each product. Supplements containing either potassium-, calcium- or magnesium citrate or any combination of these organic salts were reviewed. The total alkali load (TAL) was calculated based on the recommended daily dosage (RDD).

**Results:**

Sixteen supplements with a mean alkali powder content of 220.69 ± 111.02 g were identified. The mean magnesium content per RDD was 239.93 ± 109.16 mg. The mean potassium and median calcium content were 550 ± 325.58 mg and 280 (240) mg, respectively. Median TAL was 1220 (328.75) mg. The PRAL-lowering potential from a single RDD ranged from − 51.65 mEq to -8.32 mEq. Substantial price differences were found, and the mean price of the examined supplements was 16.67 ± 5.77 Euros. The median price for a 1 mEq PRAL-reduction was 3.01 (3.14) cents, and ranged from 0.77 cents to 10.82 cents.

**Conclusions:**

Noticeable differences between the identified alkali supplements were encountered, warranting an individual and context-specific approach in daily clinical practice.

**Supplementary Information:**

The online version contains supplementary material available at 10.1186/s40780-024-00342-0.

## Background

Western diets deficient in plant foods and abundant in processed foods exert a high potential renal acid load (PRAL) to the human kidneys [1, 2]. Several studies suggested that this might unfavorably affect cardiovascular, metabolic and bone health [3–6]. Further to that, a high PRAL was also associated with incident chronic kidney disease [1, 7], hypertension [8], and hyperuricemia [9], as well as frailty and type-2-diabetes [10, 11].

Plant-based diets were shown to reduce PRAL by incorporating plant foods that include alkali precursors on a regular basis [2, 12, 13]. Apart from a higher content of organic potassium salts and magnesium, plant-based diets lower the intake of phosphorus and preservative phosphates, which have acidifying effects on the human body [2, 14, 15].

Pharmaceutical companies nowadays sell and advertise for a variety of alkali supplements that are supposed to exert similar alkalizing effects in humans. These supplements are commonly available in German drug stores, do not require a prescription, and are often marketed as “valuable suppliers of alkali precursors” suitable for persons with an inadequate fruit and vegetable intake. As dietary/food supplements, they are “intended to supplement the general diet”, and are sold in a dosed form, for instances as powders, capsules, tablets or liquids [16, 17]. In Germany, dietary/food supplements are not medicinal products and may not have any side-effects [17–19]. They do not have to be registered with the Federal Office of Consumer Protection and Food Safety (BVL), and are subject to the German Food Supplements Regulation (NemV) [18], which specifies which vitamins and minerals may be added.

Alkali supplements contain magnesium, potassium or calcium either in the form of citrates or carbonates. Several studies suggested differences between those two supplements forms, particularly in the case of calcium [20, 21].

While calcium carbonate supplements contain more elemental calcium than calcium citrate supplements, they also require an acidic environment to dissolve, whereas calcium citrate does not. Potential differences in the resorption and in unwanted side effects fostered a controversial debate regarding the most effective calcium supplement form [22]. As for magnesium, differences in bioavailability between the different magnesium forms appear minimal [23], yet citrate-based supplements are heavily marketed in Germany for their advertised alkalizing effects on the human body [24, 25]. They are usually more expensive than carbonate-based supplements [20], and – from our personal clinical experience – less readily available.

The aims of this brief contribution were twofold: (1) to identify and comparably review the currently citrate-based dietary alkali supplements sold in Germany, and (2) to contrast the PRAL-lowering potential and other characteristics inherent to each product, focusing on additionally included minerals and vitamins which per se do not alter the PRAL.

## Methods

In October 2023, we visited three local pharmacies/drug stores in Freiburg, Germany and inquired about available citrate-based alkali supplements. In addition to that, we reviewed the available alkali supplements at several large online supplement retailers in Germany in early November 2023. Only citrate-based supplements were considered (defined as a citrate content > = 80% of the total alkali content). Supplements which included carbonates were only considered when the carbonate share was < 15% of the total content. To be considered eligible for this comparative review, supplements had to contain either potassium citrate, calcium citrate, magnesium citrate or any combination of these organic salts.

Supplement content (in g), the recommended daily dosage (RDD, in g) as well as the magnesium, potassium, sodium and calcium content of the respective supplements were reviewed. The PRAL was estimated per RDD with the following formula postulated by Remer and Manz [26]:

PRAL (mEq/RDD) = (0.49 * total protein content (g)) + (0.037 * phosphorus content (mg)) – (0.021 * potassium content (mg)) – (0.026 * magnesium content (mg)) – (0.013 * calcium content (mg)) -(0.041 * sodium content (mg)).

Sodium was added to the PRAL formula in order to increase the precision of our estimates, and because a few examined supplements contained sodium citrate (see results below), which also exerts alkalizing effects. For sodium, the conversion factor “0.041” was extracted from the work of Remer and Manz [26].

Moreover, we also calculated the so-called “total alkali load” (TAL) in mg, which is the sum of the following 4 alkalizing minerals: magnesium, potassium, sodium and calcium. The TAL (in mg) is not to be confused with the PRAL (in mEq), as the TAL reflects a (theoretical) indication of quantity.

In addition to that, we systematically captured the content of other minerals and vitamins included in each alkali supplement. Furthermore, we also reviewed whether the supplement was vegan (defined as not containing any animal-based components) or not, and whether the respective supplement was manufactured in Germany or not. In cases of doubt, we contacted the respective manufacturers by email and – if unsuccessful – by telephone. This information was collected to describe the examined dietary alkali supplements in as much detail as possible, allowing for a balanced and unbiased comparative analysis of the included products.

### Statistical analysis

STATA 14 statistical software (StataCorp. 2015. Stata Statistical Software: Release 14. College Station, TX: StataCorp LP) was used for the statistical analysis. The Shapiro-Wilk test was used to determine whether the examined data was normally distributed or not. Normally distributed variables were described with their mean ± standard deviation whereas the median and the respective interquartile range were provided for non-normally distributed variables. Box-Whisker-Plots and (stacked) bar charts were employed to graphically show the results.

## Results

A total of *n* = 16 citrate-based alkali supplements available on the German market were identified (Table 1). The mean alkali powder content of all products was 220.69 ± 111.02 g and ranged from 75 to 500 g. As such, the recommended daily dosage provided by the manufacturers also varied considerably, and ranged from 3 to 32 g per day.

**Table 1:** Alkaline supplements: recommended daily doses, mineral content and PRAL per RDD. An overview

Name	Size (g)	RDD (g)	Calcium (mg)	Potassium (mg)	Magnesium (mg)	Sodium (mg)	PRAL (mEq/RDD)	Price (€)
Dr. Jacob’s ® Basenpulver	300	4.5	270	750	185	0	-24.07	25.00
Dr. Jacob’s ® Basenpulver plus	300	6	270	750	187.5	0	-24.14	21.00
Hübner Basis Balance ® Mineralstoffe Pur	200	32	500	600	200	200	-32.50	8.95
Salus Basen-Aktiv ® Mineralstoff-Kräuterextrakt-Pulver	90	3	240	300	112	0	-12.33	17.95
Bonemis® Basenpulver (Citrate)	500	10.5	800	1500	375	0	-51.65	18.90
Basica Vital® pur, Basenpulver	81	8.1	550	350	150	375	-33.78	15.95
Basica Vital®, Basisches Granulat	200	32	550	350	150	375	-33.78	10.95
MADENA Basenpulver BasenCitrate Pur ®	216	8.2	260	600	360	0	-25.34	16.95
MADENA Basenpulver BasenCitrate Pur Komplex ®	240	10	260	600	360	0	-25.34	22.95
Sanotact ® pH-Basenbalance Pulver	200	24	400	400	187.5	0	-18.48	3.99
Nutrition-Plus Germany e.K. Basenpulver	224	5.6	260	600	360	0	-25.34	14.99
NUTRITHEKE Green Line Basencitrate ohne Zucker	160	7.6	280	600	375	0	-25.99	15.95
Naturafit Basencitrate zuckerfrei	160	7.6	280	600	375	0	-25.99	16.95
IHLEVITAL Basenpulver BasenDepot	75	3	320	0	160	0	-8.32	22.50
BioPräp Basenpulver vegan	200	16	248	200	61.8	72	-11.98	10.80
Naturalie ® Basenkomplex - Basenpulver mit Citraten	385	7	500	600	240	100	-29.44	22.99

RDD = Recommended Daily Dosage, PRAL = Potential Renal Acid Load. Calcium, potassium, magnesium and sodium in mg/RDD. PRAL in mEq/RDD.

**Fig. 1:**
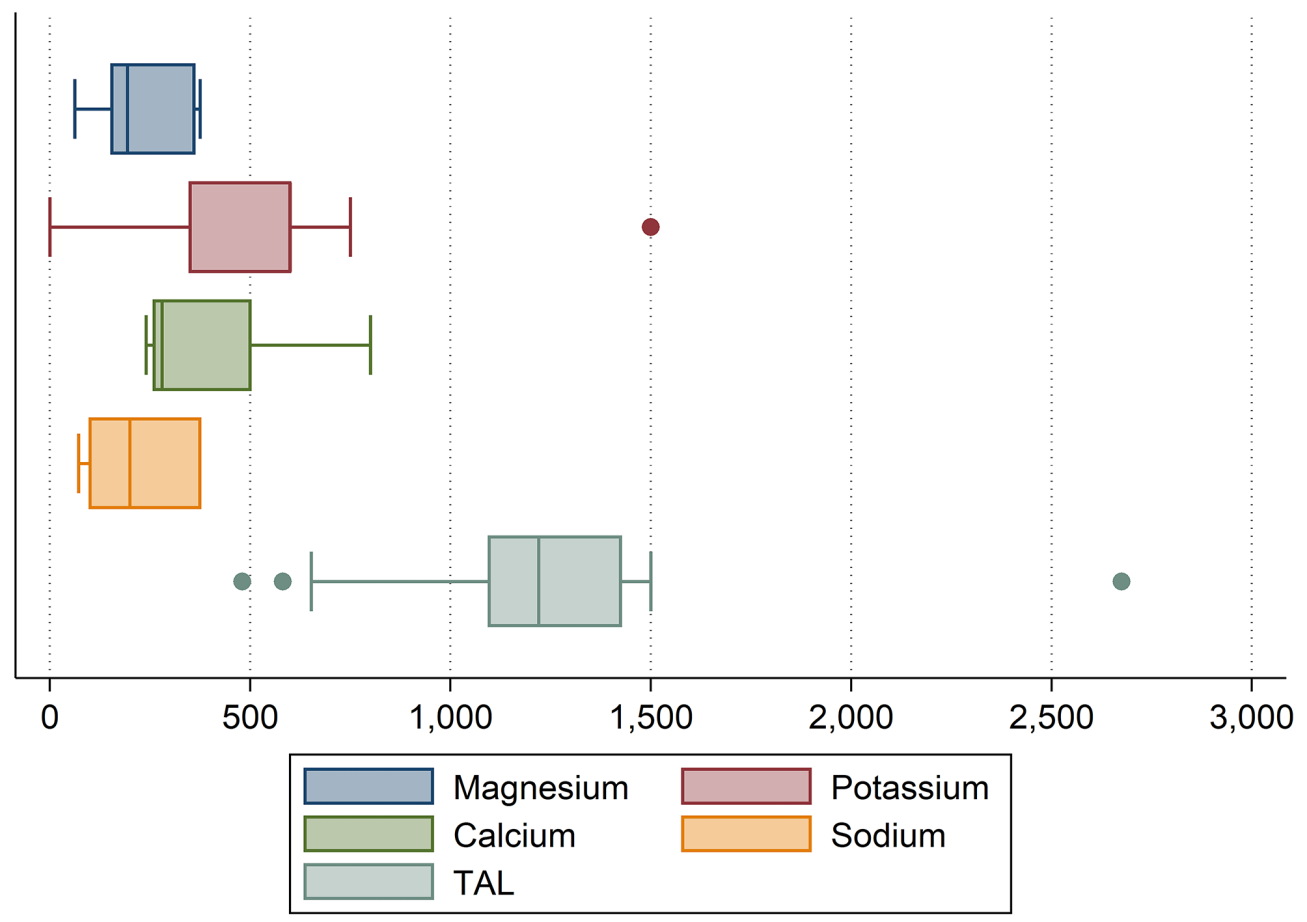
Boxplots showing the distribution of the magnesium, calcium, sodium and potassium content of the examined alkali supplements. Magnesium, potassium and calcium and sodium in mg per daily recommended dose. The sum of these 4 minerals reflects the “total alkali load” of the examined items. TAL = Total Alkali Load. Based on *n* = 16 alkali supplements. Note: only *n* = 15 of all supplements (93.75%) contained potassium and *n* = 5 (31.25%) supplements contained sodium

Table 1 displays the potassium, magnesium, sodium and calcium content of the examined products. None of the examined products contained (acidifying) protein and/or phosphorus. As shown in Fig. 1, large differences were found with regard to the mineral content of the examined supplements when comparing daily recommend dosages.

The mean magnesium content per daily recommended dosage was 239.93 ± 109.16 mg. The mean potassium and median calcium content were 550 ± 325.58 mg and 280 (240) mg, respectively. Only *n* = 5 items (31.25%) contained sodium. The mean amount of sodium contained was 224.4 ± 145.48 mg per daily recommend dosage.

**Fig. 2:**
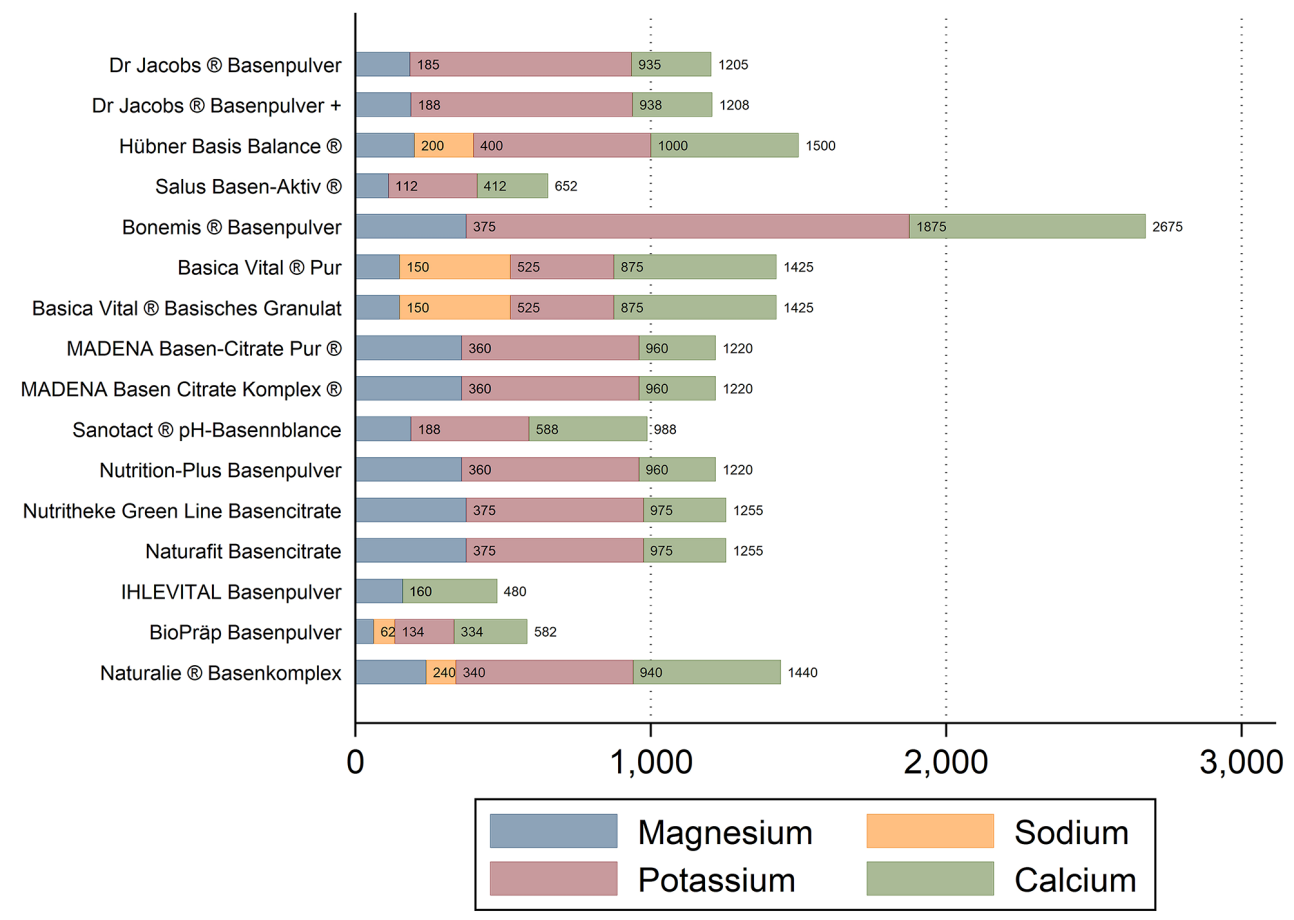
Total alkali load of the examined alkali supplements: an overview. Magnesium, sodium, potassium and calcium in mg per daily recommended dose. The sum of these 4 minerals reflects the “total alkali load” of the examined items. Based on *n* = 16 alkali supplements

Subsequently, large differences were also found for the TAL (total alkali load) of the examined supplements (Fig. 2). The median TAL was 1220 (328.75) mg and ranged from 480 mg to 2675 mg per daily recommended dosage.

The PRAL-lowering potential from a single RDD ranged from − 51.65 mEq (Bonemis® Basenpulver) to -8.32 mEq (IHLEVITAL Basenpulver BasenDepot). The mean PRAL reduction achieved with a manufacturer-recommended daily dosage was − 25.53 ± 10.30 mEq.

**Fig. 3:**
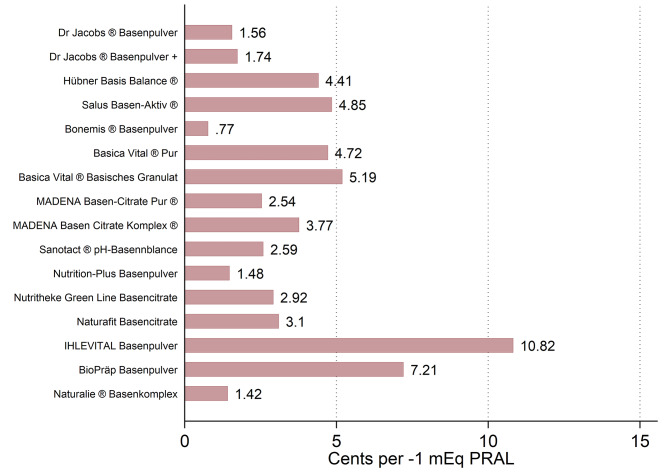
Costs in cents (€) per 1 mEq PRAL reduction: an overview. Costs shown in cents per 1 mEq PRAL reduction. Based on *n* = 16 alkali supplements

Substantial price differences were found with regard to all supplements. The mean price of the examined alkali supplements (not size-standardized) was 16.67 ± 5.77 Euros. For a better comparison, we also calculated the price that a customer pays for each 1 mEq PRAL reduction when purchasing the respective dietary supplement (Fig. 3). The median price for each − 1 mEq was 3.01 (3.14) cents, and ranged from 0.77 cents to 10.82 cents overall.

Only *n* = 3 dietary supplements did not contain additional vitamins or minerals (18.75%), whereas the remaining *n* = 13 items (81.25%) were enriched with additional compounds (Table 2). *N* = 4 supplements (25%) contained silicon and *n* = 13 items (81.25%) contained zinc in a median dosage of 5 mg per daily recommended dose. Vitamin C was found in *n* = 2 items (12.5%) and Vitamin D was found in *n* = 5 supplements (31.25%) in an average dose of 444 ± 350.71 IU. Copper was found in *n* = 5 of the examined supplements (31.25%) and in an average dose of 0.72 ± 0.26 mg. Chromium and Molybdenum were found in *n* = 6 items (37.5%), each.

**Table 2:** title: Alkaline supplements: sodium, trace element and vitamin content

Name	Other minerals / vitamins included	Si	Zn	VB1	VC	VD	VK2	Fe	Cu	Mn	Se	Cr	Mo	B	I
Dr. Jacob’s ® Basenpulver	Yes	20	2.5	0.7		100	-	-	-	-	-	-	-	-	-
Dr. Jacob’s ® Basenpulver plus	Yes	23	2	0.42	80	100	-	-	-	-	-	-	-	-	-
Hübner Basis Balance ® Mineralstoffe Pur	Yes	-	5	-	-	-	-	5	1	1	30	60	80	-	-
Salus Basen-Aktiv ® Mineralstoff-Kräuterextrakt-Pulver	Yes	-	5	-	-	-	-	-	-	-	-	-	-	-	-
Bonemis® Basenpulver (Citrate)	No	-	-	-	-	-	-	-	-	-	-	-	-	-	-
Basica Vital® pur, Basenpulver	Yes	-	5	-	-	-	-	-	-	-	30	40	50	-	-
Basica Vital®, Basisches Granulat	Yes	-	5	-	-	-	-	5	1	-	30	40	50	-	-
MADENA Basenpulver BasenCitrate Pur ®	Yes	-	5	-	-	800	-	-	-	-	-	-	-	-	-
MADENA Basenpulver BasenCitrate Pur Komplex ®	Yes	60	5	-	200	800	70	-	0.5	1	50	50	50	3	-
Sanotact ® pH-Basenbalance Pulver	Yes	28	5	-	-	-	-	7	0.5	1	27.5	20	25	-	-
Nutrition-Plus Germany e.K. Basenpulver	Yes	-	5	-	-	400	-	-	-	-	-	-	-	-	-
NUTRITHEKE greenline Basencitrate ohne Zucker	No	-	-	-	-	-	-	-	-	-	-	-	-	-	-
Naturafit Basencitrate zuckerfrei	No	-	-	-	-	-	-	-	-	-	-	-	-	-	-
IHLEVITAL Basenpulver BasenDepot	Yes	-	3	-	-	-	-	-	-	-	-	-	-	-	-
BioPräp Basenpulver vegan	Yes	-	3	-	-	-	-	3.2	0.62	-	55	37	50		62
Naturalie ® Basenkomplex - Basenpulver mit Citraten	Yes	-	10	-	-	-	-	-	-	-	-	-	-	-	-

Si = Silicum (in mg); Zn = Zinc (in mg); VB1 = Vitamin B1 (in mg); VC = Vitamin C (in mg); VD = Vitamin D (in IU); VK2 = Vitamin K2 (in µg); Fe = Iron (in mg); Cu = Copper (in mg); Mn = Manganese (in mg); Se = Selenium content (in µg); Cr = Chromium content (in µg); Mo = Molybdenum content (in µg); Boron content (in mg); I = Iodine content (in µg)

*N* = 2 supplements (12.5%) were not vegan (e.g. without any content of animal origin), whereas the remaining *n* = 14 items (87.5%) were declared vegan by the respective manufacturers. *N* = 15 items were produced in Germany; in *n* = 1 cases the origin was not ascertainable even after contacting the respective manufacturer (“Hübner Basis Balance ® Mineralstoffe Pur”).

## Discussion

Similar to a more recent US-based study, we reviewed the currently available over-the-counter citrate-based alkali supplements sold in Germany [27]. Noticeable differences between the identified supplements were encountered, both with regard to the mineral content as well as with the PRAL-lowering potential of each product.

Prospective clinical studies investigating the effects of such supplements rich in alkaline minerals on the acid-base balance in humans are generally scarce and limited to a handful of trials [28, 29]. Vormann et al. hypothesised that a latent chronic acidosis subsequent to a high PRAL might contribute to low back pain (LBP), and conducted an open prospective study in *n* = 82 patients with chronic LBP who received 30 g of a lactose based alkaline multimineral supplement (Basica®) daily for a period of 4 weeks [28]. Pain symptoms were assessed with the “Arhus low back pain rating scale” (ARS), and mean ARS scores dropped significantly by 49% (-20 points in total) after 4 weeks of supplementation. König et al. enrolled *n* = 25 (15 females; 10 males) participants in a study investigating the effects of a multimineral alkali supplement (MMS) on acute and chronic regulation of acid-base balance with the pH of blood, urine and saliva as potential surrogate markers [29]. Blood and urinary pH significantly increased in this uncontrolled study. Following a longer supplementation period, both the increase in urinary pH in the morning and in the evening occurred within 24 h. Compared to pH values without the MMS, average pH in urine was more than 10% higher in the morning and approximately 5% higher in the evening.

Moseley et al. suggest that potassium citrates may also improve skeletal health [30]. The authors conducted a randomized, double blind and placebo controlled study in 52 elderly women and men with a mean age 65.2 ± 6.2 years. Participants were randomly assigned to one of the following regimens: potassium citrate 60 mmol per day, 90 mmol per day or placebo. Bone turnover markers, net acid excretion and calcium balance were examined at baseline and after a half year. Notably, net acid excretion decreased significantly in both treatment groups when compared to the place. The regular supplement intake resulted in a complete neutralization of the participants’ dietary acid load. Favorable findings were also reported by Granchi et al. in a 2018 study, which also employed a potassium citrate supplement in osteopenic women [31].

**Fig. 4:**
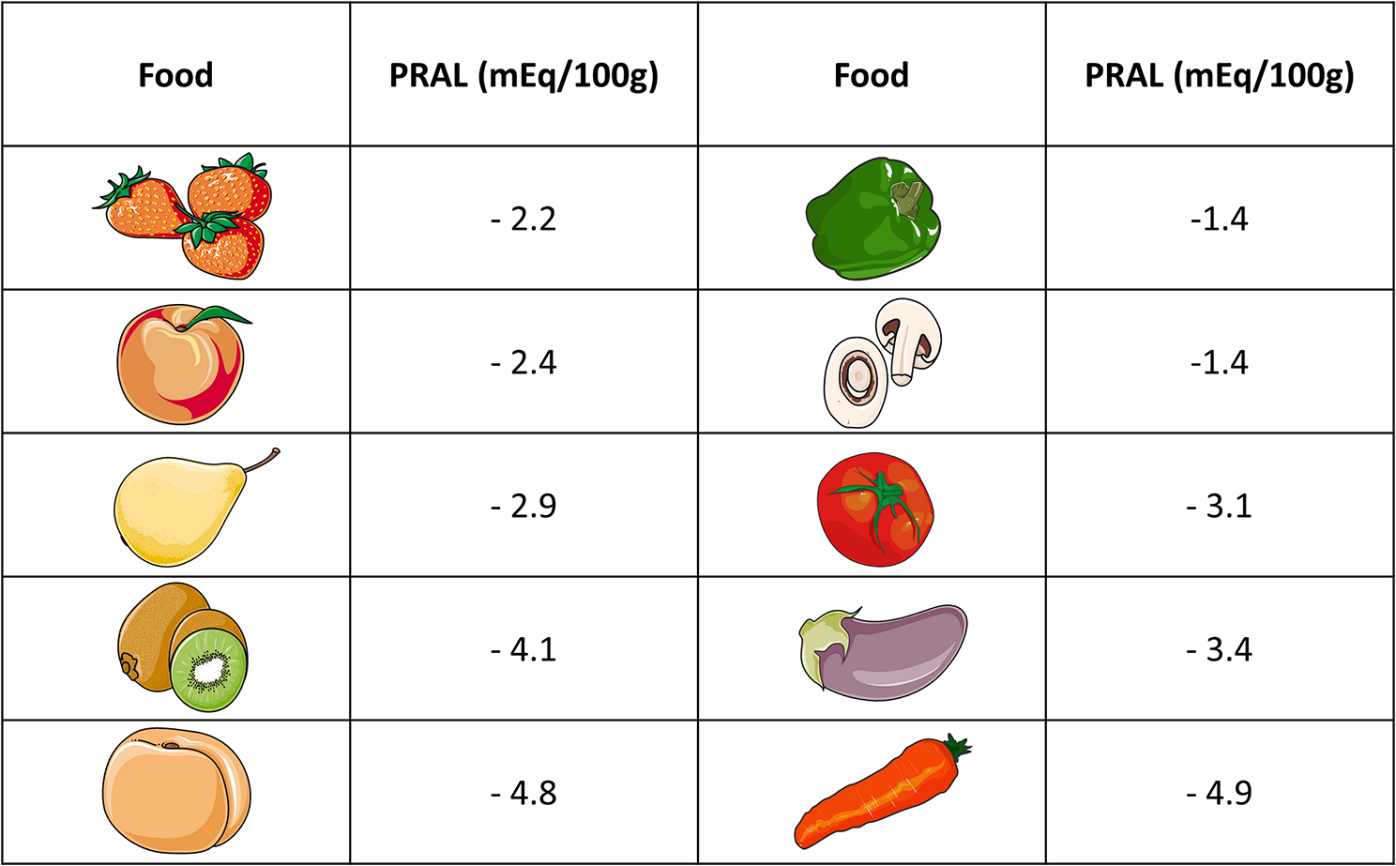
Potential renal acid load of commonly consumed alkalizing vegetables. Based on [26], images modified from Servier Medical Art database by Servier (Creative Commons 3.0; https://smart.servier.com/)

While large-scale randomized-controlled clinical trials are scarce in this field of study for endpoints unrelated to the human kidneys or bone health, alkali supplements might serve several purposes. From our personal clinical perspective, they might be a helpful tool to decrease PRAL in individuals with an otherwise high dietary acid load, particularly when transitioning towards a more plant-based diet. For a quantitative comparison, Fig. 4 summarizes the PRAL values of some commonly consumed alkalizing fruits and vegetables, based on data obtained from Remer and Manz [26].

A clear reservation must be made that such supplements do not qualify as fruit and vegetable substitutes, and may not replace a healthy diet. Fruits and vegetables contain numerous health promoting substances, including carotenoids, phenolic antioxidants, and anthocyanidin, which all have proven benefits to human health [32].

In this context, a reduction in dietary acid load has been shown to reduce kidney injury in patients with moderately reduced eGFR (glomerular filtration rate) due to hypertensive nephropathy, and fruits and vegetables were comparable to sodium bicarbonate in an investigation by Goraya et al. [33]. Notably, newer studies by the same authors highlighted the additional benefits of fruits and vegetables over oral alkali supplementation [34].

Here, we reviewed citrate-based supplements available in Germany and found noticeable differences between the different supplements. Not designed as a head-to-head comparison, we intentionally focused on a descriptive analysis and do *not* recommend one supplement over another supplement. To the contrary, we encourage the reader to carefully review the unique characteristics of each examined supplement described here, and to consider whether an enrichment with other minerals might be suitable in a particular clinical situation or not. This might be of particular importance because many supplements contained additional nutrients that may not necessarily decrease PRAL but are marketed under different purposes. A common example is zinc, which is added to almost all examined supplements (Table 2), and which - according to the PRAL formula by Remer and Manz - does not alter PRAL. We can only guess why additional nutrients were added to the examined supplements and supposed that they were included for marketing purposes (e.g., because an optimal zinc status is associated with a healthy immune system). Whether necessary is subject to a controversial debate and beyond the scope of this paper. Yet, we deemed it important to systematically capture these nutrients and vitamins added to the examined dietary supplements even when they do not add to their PRAL-lowering potential.

Whether such supplements enriched with additional vitamins and nutrients may be helpful in a particular clinical situation *cannot* be answered with this cross-sectional study design and is beyond the scope of the paper. Likewise, we did not review supplement characteristics such as flavor, taste or consistency.

As such, this short contribution has strengths and weaknesses that warrant a careful consideration. Entirely descriptive, we do *not* recommend a particular supplement over another supplement, but provide the reader with a list of potentially available citrate-based dietary alkali supplements in Germany. Although we carefully screened multiple online pharmacies and local drug stores to capture as many products as possible, we may not guarantee a complete list of products. In addition, we only considered food/dietary supplements for this analysis; medicinal products and drugs that warrant a prescription from a physician for a specific medical indication were not considered here. Finally, we acknowledge that the primary source of each examined product (e.g. rock (dolomite, limestone), animal (oyster shellfish powder, coral), plant (plant and wood ash) was not ascertainable from the available data. Although many products were declared “vegan” and as such contained no ingredient of animal origin, we may not provide additional details here.

The authors wish to emphasize that they declare no conflicts of interest and no supplement was reviewed more favorably than another one.

## Conclusions

Despite some limitations, we provide the first comprehensive overview of the available citrate-based dietary alkali supplements in Germany. The identified noticeable differences between the examined supplements warrant an individual and context-specific approach in daily clinical practice when recommending alkali supplements.

### Electronic supplementary material

Below is the link to the electronic supplementary material.

Supplementary Material 1

## Data Availability

The dataset used and analyzed during the current study is available from the corresponding author on reasonable request.

## Abbreviations

PRAL: Potential Renal Acid Load
RDD: Recommended Daily Dosage
TAL: Total Alkali Load

## References

[1] Tariq A, Chen J, Yu B, Boerwinkle E, Coresh J, Grams ME (2022). Metabolomics of Dietary Acid load and incident chronic kidney disease. J Ren Nutr.

[2] Storz MA, Ronco AL (2022). Reduced dietary acid load in U.S. vegetarian adults: results from the National Health and Nutrition Examination Survey. Food Sci Nutr.

[3] Sanz JM, Sergi D, Colombari S, Capatti E, Situlin R, Biolo G et al. Dietary Acid Load but Not Mediterranean Diet Adherence Score Is Associated With Metabolic and Cardiovascular Health State: A Population Observational Study From Northern Italy. Frontiers in Nutrition [Internet]. 2022 [cited 2023 Dec 15];9. https://www.frontiersin.org/articles/10.3389/fnut.2022.828587. 10.3389/fnut.2022.828587 PMC9087734 35558749

[4] Armin M, Heidari Z, Askari G, Iraj B, Clark CCT, Rouhani MH (2023). The effect of a low renal acid load diet on blood pressure, lipid profile, and blood glucose indices in patients with type 2 diabetes: a randomized clinical trial. Nutr J.

[5] Mangano KM, Walsh SJ, Kenny AM, Insogna KL, Kerstetter JE (2014). Dietary acid load is associated with lower bone mineral density in men with low intake of dietary calcium. J Bone Min Res.

[6] Wieërs MLAJ, Beynon-Cobb B, Visser WJ, Attaye I. Dietary acid load in health and disease. Eur J Physiol. 2024;476:427–433. 10.1007/s00424-024-02910-7 10.1007/s00424-024-02910-7 PMC11006742 38282081

[7] Yurtdaş Depboylu G, Kaner G, Özdemir Şimşek Ö, Turan KN, Kasap Demir B (2023). Dietary acid load in children with chronic kidney disease: its association with nutritional status and health-related quality of life. Pediatr Nephrol.

[8] Lin F, Zhang M, Wang R, Sun M, Zhang Z, Qiao Y (2023). Association between Dietary Acid load and hypertension in Chinese adults: analysis of the China Health and Nutrition Survey (2009). Nutrients.

[9] Seifi N, Bahari H, Nosrati M, Koochakpoor G, Alizadeh Hassani Z, Rastegarmoghadam-Ebrahimian A et al. Higher dietary acid load is associated with the risk of hyperuricemia. Int Urol Nephrol [Internet]. 2023 Dec 10 [cited 2023 Dec 15]; 10.1007/s11255-023-03876-8. 10.1007/s11255-023-03876-8 38072898

[10] Kataya Y, Murakami K, Kobayashi S, Suga H, Sasaki S, Three-generation Study of Women on Diets and Health Study Group (2018). Higher dietary acid load is associated with a higher prevalence of frailty, particularly slowness/weakness and low physical activity, in elderly Japanese women. Eur J Nutr.

[11] Ozturk EE, Yildiz H (2023). Examining dietary acid load in individuals with type 2 diabetes: a case-control study. Eur Rev Med Pharmacol Sci.

[12] Betz MV, Penniston KL (2023). Primary contributors to Dietary Acid load in patients with Urolithiasis. J Ren Nutr.

[13] Kahleova H, McCann J, Alwarith J, Rembert E, Tura A, Holubkov R (2021). A plant-based diet in overweight adults in a 16-week randomized clinical trial: the role of dietary acid load. Clin Nutr ESPEN.

[14] Storz MA, Ronco AL, Hannibal L (2022). Observational and clinical evidence that plant-based nutrition reduces dietary acid load. J Nutritional Sci.

[15] Ekmeiro-Salvador JE, Storz MA (2023). The impact of plant-based diets on Dietary Acid load Metrics in Venezuela: a cross-sectional study. Nutrients.

[16] Health assessment of. food supplements - BfR [Internet]. [cited 2024 Apr 5]. https://www.bfr.bund.de/en/health_assessment_of_food_supplements-736.html.

[17] BVL - Nahrungsergänzungsmittel vs. Arzneimittel [Internet]. [cited 2024 Apr 5]. https://www.bvl.bund.de/DE/Arbeitsbereiche/01_Lebensmittel/03_Verbraucher/04_NEM/01_NEM_Arzneimittel/NEM_Arzneimittel_node.html.

[18] NemV - nichtamtliches Inhaltsverzeichnis [Internet]. [cited 2024 Apr 5]. https://www.gesetze-im-internet.de/nemv/index.html.

[19] Bundesinstitut für Risikobewertung. Nährstoffversorgung? Teller statt Tablette! [cited 2024 Apr 5]. https://www.bfr.bund.de/cm/343/fragen-und-antworten-zu-nahrungsergaenzungsmitteln.pdf.

[20] Naciu AM, Tabacco G, Bilezikian JP, Santonati A, Bosco D, Incognito GG (2022). Calcium citrate Versus Calcium Carbonate in the management of chronic hypoparathyroidism: a Randomized, Double-Blind, crossover clinical trial. J Bone Miner Res.

[21] Wark JD, Nowson C. Calcium supplementation: the bare bones. Australian Prescriber [Internet]. 2003 Dec 1 [cited 2023 Dec 15];26(6). https://australianprescriber.tg.org.au/articles/calcium-supplementation-the-bare-bones.

[22] Pischel P, Conrad I, Zwingers F, Rzymski T, Opala P (2016). The bioavailability of calcium in the form of pyruvate, carbonate, citrate–malate in healthy postmenopausal women. Eur Food Res Technol.

[23] Schuchardt JP, Hahn A (2017). Intestinal absorption and factors influencing bioavailability of Magnesium-An Update. Curr Nutr Food Sci.

[24] Dr. Jacob’s Medical GmbH [Internet]. [cited 2023 Dec 15]. Dr. Jacob’s Basenpulver 300 g online bestellen. https://drjacobs-shop.de/dr.-jacob-s-basenpulver-300-g/nbvpd.

[25] Basica® Pur –. reines Basenpulver | Basica® [Internet]. [cited 2023 Dec 15]. https://www.basica.com/de/Produkte/Fuer-innere-Balance/Basica-Pur_product_2479.

[26] Remer T, Manz F (1995). Potential renal acid load of foods and its influence on urine pH. J Am Diet Assoc.

[27] Dai JC, Maalouf NM, Hill K, Antonelli JA, Pearle MS, Johnson BA (2023). Alkali Citrate Content of Common Over-the-counter and Medical Food supplements. J Endourol.

[28] Vormann J, Worlitschek M, Goedecke T, Silver B (2001). Supplementation with alkaline minerals reduces symptoms in patients with chronic low back pain. J Trace Elem Med Biol.

[29] König D, Muser K, Dickhuth HH, Berg A, Deibert P (2009). Effect of a supplement rich in alkaline minerals on acid-base balance in humans. Nutr J.

[30] Moseley K, Weaver C, Appel L, Sebastian A, Sellmeyer DE (2013). Potassium citrate supplementation results in sustained improvement in calcium balance in older men and women. J Bone Min Res.

[31] Granchi D, Caudarella R, Ripamonti C, Spinnato P, Bazzocchi A, Massa A (2018). Potassium citrate supplementation decreases the biochemical markers of bone loss in a Group of Osteopenic women: the results of a Randomized, Double-Blind, placebo-controlled pilot study. Nutrients.

[32] Wallace TC, Bailey RL, Blumberg JB, Burton-Freeman B, Chen C y., Crowe-White O et al. KM,. Fruits, vegetables, and health: A comprehensive narrative, umbrella review of the science and recommendations for enhanced public policy to improve intake. Critical Reviews in Food Science and Nutrition. 2020;60(13):2174–211. 10.1080/10408398.2019.1632258 31267783

[33] Goraya N, Simoni J, Jo C, Wesson DE (2012). Dietary acid reduction with fruits and vegetables or bicarbonate attenuates kidney injury in patients with a moderately reduced glomerular filtration rate due to hypertensive nephropathy. Kidney Int.

[34] Goraya N, Munoz-Maldonado Y, Simoni J, Wesson DE (2019). Fruit and Vegetable treatment of chronic kidney disease-related metabolic acidosis reduces Cardiovascular Risk Better than Sodium bicarbonate. Am J Nephrol.

