# Supplementary material for: Citrate-based dietary alkali supplements available in Germany: an overview
Source: J Pharm Health Care Sci. 2024 May 10;10:22. doi: 10.1186/s40780-024-00342-0 (PMC11084099; doi:10.1186/s40780-024-00342-0)
Supplement: Supplementary file 1 — Supplementary Material 1 [file 40780_2024_342_MOESM1_ESM.pdf]

UNIVERSITÄTSKLINIKUM FREIBURG
Klinik für Innere Medizin II
Haus Frerichs, Hugstetterstr. 55, 79106 Freiburg

Journal of Pharmaceutical Health Care and Sciences
Editorial Office

Prof. Dr. Muraki
Kyoto Pharmaceutical University, Japan

Klinik für Innere Medizin II

Ärztlicher Direktor:
Prof. Dr. med. Robert Thimme

Uni-Zentrum Naturheilkunde
Leiter: Prof. Dr. med. Roman Huber

Haus Frerichs, Hugstetterstr. 55, 79106 Freiburg
Tel 0761 270-82010
Fax 0761 270-83230
E-Mail: roman.huber@uniklinik-freiburg.de
www.uni-zentrum-naturheilkunde.de

30 April 2024

Cover letter for our revised submission to the Journal of Pharmaceutical Health Care and Sciences: JPHC-D-24-00026R1

Dear Professor Dr. Muraki, dear Professor Dr. Saito

It is with great excitement that we resubmit to you a revised version of our original article entitled “**Citrate-Based Dietary Alkali Supplements Available in Germany: An Overview**” for consideration of publication in **JPHCS**. This manuscript has been assigned the number JPHC-D-24-00026.R1 and was formerly submitted under the title “*Over-The-Counter Citrate-Based Alkali Supplements Available in Germany: An Overview*”.

We would like to thank you and the involved Reviewers again very much for careful and thorough reading of this manuscript and for the thoughtful comments and constructive suggestions, which helped us to improve the quality of this article. All requested changes have been clearly marked in yellow and blue color in the revised manuscript.

More specifically, we changed the paragraph order in the methods section, following the comments of Reviewer #1. Only minor revisions were applied, thus the main message behind the manuscript remains unaltered. Please find our summary below.

The Potential Renal Acid Load (PRAL) is a marker of recent clinical and epidemiological interest, indicating the net effects of foods on the human acid-base equilibrium. Fruits and vegetables are abundant in alkali precursors and thus effectively reduce the PRAL from diet.

Dietary alkali supplements are supposed to exert similar alkalizing effects on the human body, and are thus consumed and sold widely.

Here, we comparatively reviewed the currently available over-the-counter citrate-based alkali supplements sold in Germany with a special focus on their mineral content, their PRAL-lowering potential and other characteristics inherent to each product. Supplements containing either potassium-, calcium- or magnesium citrate or any combination of these organic salts were reviewed. The total alkali load (TAL) was calculated based on recommended daily dosages (RDD). Our findings revealed an inhomogeneous picture, which can be summarized as follows:
Sixteen supplements with a mean alkali powder content of 220.69 ± 111.02 g were identified. The mean magnesium content per RDD was 239.925 ± 109.164 mg. The mean potassium and median calcium content were 550 ± 325.58 mg and 280 (240) mg, respectively. Median TAL was 1220 (328.75) mg. The PRAL-lowering potential from a single RDD ranged from -51.65 mEq to -8.32 mEq. Substantial price differences were found, and the mean price of the examined supplements was 16.67 ± 5.77 Euros. The median price for a 1 mEq PRAL-reduction was 3.01 (3.12) cents, and ranged from 0.77 cents to 10.82 cents.

Based on these data, we identified noticeable differences between the examined supplements, warranting an individual and context-specific approach in daily clinical practice. In our submission, we discuss the potential usage and limitations of the examined supplements. To the best of our knowledge, we are the first group to comparatively analyze the available citrate-based alkali supplements in Germany. Our data might be of great help for clinicians, healthcare professionals and patients interested in alkalizing their diet. Our detailed comparative analysis might be a good orientation for these groups to make context-specific decisions and to choose the most appropriate supplement.

We believe that this original article may align well with the aims and scope of your journal. Our comparative study discusses an interesting topic at the intersection of supplementation, pharmacology, nutrition and the acid-base equilibrium. It could thus be of interest for your broad readership. Based on our experience in the field of dietary acid load, we are convinced that the paper will make an important contribution and has the potential for multiple citations.

We confirm that this work is original and has not been published elsewhere, nor is it currently under consideration of publication elsewhere. The publication has been approved by all authors. We have no conflicts of interest to disclose.

Thank you for your consideration of this manuscript.

Sincerely yours,

Maximilian Storz

Maximilian Storz (on behalf of Prof. Dr. Ronco)
